# Supplementary material for: Sweet Potato (Ipomoea batatas L.) Phenotypes: From Agroindustry to Health Effects
Source: Foods. 2022 Apr 6;11(7):1058. doi: 10.3390/foods11071058 (PMC8997864; doi:10.3390/foods11071058)
Supplement: Supplementary file 1 [file foods-11-01058-s001.zip › Table S1.pdf]

Supplementary Material (Table S1) - Agricultural outlook 1999-2019: Sweet Potatoes (Complete FAOSTAT Data)

	1999			2009			2019		
Country	A (Ha)	P (Tons)	Yield (Hg/Ha)	A (Ha)	P (Tons)	Yield (Hg/Ha)	A (Ha)	P (Tons)	Yield (Hg/Ha)
Réunion	15	300	200,000	--	--	--	--	--	--
Martinique	120	1,170	97,500	--	--	--	--	--	--
Guadeloupe	390	4,267	109,410	--	--	--	--	--	--
Greece	339	6,250	184,366	173	3,522	203,584	--	--	--
Italy	1,275	10,231	80,243	439	7,771	177,016	--	--	--
Spain	1,267	20,364	160,726	1,443	24,838	172,128	--	--	--
Portugal	2,668	23,687	88,782	1,758	23,490	133,618	--	--	--
Bhutan	--	--	--	104	105	10,096	25	48	19,200
Botswana	--	--	--	--	--	--	300	3,000	100,000
Malawi	--	--	--	--	--	--	368,927	5,908,989	160,167
Niue	14	247	176,429	15	251	167,333	16	251	156,875
Suriname	16	196	122,500	7	98	140,000	66	888	134,545
Belize	25	107	42,800	4	32	80,000	18	86	47,778
Antigua and Barbuda	26	153	58,846	43	269	62,558	44	289	65,682
Maldives	29	48	16,552	15	12	8,000	2	2	10,000
Guatemala	30	200	66,667	10	65	65,000	17	109	64,118
Yemen	33	318	96,364	45	407	90,444	42	352	83,810
Brunei Darussalam	35	180	51,429	41	252	61,463	36	284	78,889
Trinidad and Tobago	35	222	63,429	138	271	19,638	314	967	30,796
Mauritius	35	400	114,286	77	950	123,377	40	414	103,500
Cook Islands	46	1,212	263,478	18	480	266,667	6	175	291,667
Costa Rica	50	150	30,000	85	250	29,412	201	601	29,900
Saint Kitts and Nevis	50	170	34,000	26	230	88,462	40	306	76,500
Saint Lucia	50	480	96,000	42	397	94,524	23	530	230,435
Bahamas	68	259	38,088	386	1,332	34,508	592	2,080	35,135
Grenada	72	200	27,778	46	128	27,826	2,821	7,886	27,955
El Salvador	83	525	63,253	104	636	61,154	132	760	57,576
Honduras	120	763	63,583	1,090	7,000	64,220	2,843	16,984	59,740
New Caledonia	167	490	29,341	595	689	11,580	626	626	10,000
Palestine	167	5,037	301,617	95	2,500	263,158	67	1,832	273,433
Barbados	220	3,500	159,091	41	888	216,585	91	2,093	230,000
Tonga	249	3,080	123,695	572	6,352	111,049	651	6,483	99,585
Puerto Rico	320	2,526	78,938	298	2,633	88,356	58	492	84,828
Ecuador	337	1,913	56,766	1,900	3,600	18,947	2,240	3,922	17,509
Dominica	436	1,787	40,986	410	2,086	50,878	380	2,385	62,763
Fiji	477	5,224	109,518	357	4,894	137,087	901	9,053	100,477
Micronesia (Federated States of)	530	3,075	58,019	543	3,094	56,980	561	3,108	55,401
Somalia	540	5,400	100,000	772	7,608	98,549	868	8,626	99,378
Morocco	545	7,290	133,761	695	11,882	170,964	457	9,366	204,945
Cabo Verde	720	3,800	52,778	450	7,700	171,111	195	3,395	174,103
Australia	800	22,000	275,000	1,693	42,460	250,797	2,168	78,910	363,976
Pakistan	825	9,268	112,339	1,046	11,951	114,254	1,688	15,963	94,568
Israel	894	8,600	96,197	3,444	32,237	93,603	2,417	35,032	144,940
New Zealand	1,000	17,000	170,000	1,036	12,400	119,691	1,202	12,050	100,250
Chile	1,077	7,382	68,542	1,303	11,303	86,746	1,410	18,436	130,752
Eswatini	1,264	2,300	18,196	1,358	2,500	18,409	1,285	2,458	19,128
Congo	1,350	9,000	66,667	976	6,853	70,215	1,306	9,395	71,937
Guyana	1,354	2,990	22,083	93	2,310	248,387	511	14,151	276,928
Saint Vincent and the Grenadines	1,363	2,181	16,001	2,300	2,800	12,174	253	2,500	98,814
Venezuela (Bolivarian Republic of)	1,530	13,928	91,033	750	8,000	106,667	1,665	17,336	104,120
Jamaica	1,543	24,970	161,828	2,004	34,229	170,803	2,456	43,188	175,847
Gabon	1,600	2,800	17,500	1,665	3,124	18,763	2,119	4,227	19,948
Liberia	1,800	18,000	100,000	2,016	20,895	103,646	2,136	24,455	114,490
Togo	2,036	9,291	45,634	597	3,989	66,817	2,375	8,672	36,514
Burkina Faso	2,058	17,294	84,033	6,419	81,499	126,965	5,907	66,383	112,380
Mauritania	2,077	2,066	9,947	2,301	2,573	11,182	2,826	5,081	17,979
Zimbabwe	2,202	4,813	21,857	9,235	23,285	25,214	12,512	35,400	28,293
Comoros	2,284	5,141	22,509	2,807	6,503	23,167	3,394	8,001	23,574
Malaysia	2,300	25,000	108,696	1,309	13,495	103,094	3,317	56,323	169,801
Niger	2,400	49,500	206,250	3,042	48,742	160,230	6,233	173,171	277,829
Mexico	2,458	47,465	193,104	2,313	44,052	190,454	3,104	59,358	191,231
Senegal	3,034	26,538	87,469	1,500	60,000	400,000	2,316	89,397	385,997
Bolivia (Plurinational State of)	3,100	13,600	43,871	1,616	7,551	46,726	1,070	4,487	41,935
Timor-Leste	3,500	14,000	40,000	4,807	12,790	26,607	1,375	2,862	20,815
Mali	4,407	46,779	106,147	12,381	236,759	191,228	19,807	312,477	157,761
Solomon Islands	5,200	74,000	142,308	6,700	96,000	143,284	7,614	107,894	141,705
Sudan (former)	5,400	116,000	214,815	9,660	216,000	223,602	25,130	253,628	100,926
Myanmar	5,414	30,585	56,492	7,200	59,000	81,944	6,537	62,631	95,810
Uruguay	6,000	60,000	100,000	7,600	72,000	94,737	8,805	80,444	91,362
Sierra Leone	7,547	34,200	45,316	38,724	176,969	45,700	18,675	178,753	95,718
Sri Lanka	8,380	51,600	61,575	6,000	47,270	78,783	3,255	35,607	109,392
Dominican Republic	8,558	52,952	61,874	6,086	47,147	77,468	6,914	54,714	79,135
Cambodia	9,322	32,516	34,881	9,283	78,891	84,984	8,063	42,473	52,676
Paraguay	10,085	79,365	78,696	5,120	44,511	86,936	5,087	50,520	99,312
Egypt	10,520	253,053	240,545	12,517	357,266	285,425	14,165	454,041	320,537
Benin	10,885	68,847	63,249	11,471	63,695	55,527	10,279	68,323	66,469
Equatorial Guinea	11,735	36,000	30,677	17,582	81,244	46,209	19,829	124,115	62,593
Lao People's Democratic Republic	13,050	80,600	61,762	18,131	171,147	94,395	5,720	114,885	200,848
Peru	14,552	244,671	168,136	16,006	262,724	164,141	16,764	301,344	179,757
South Africa	14,774	52,000	35,197	21,000	63,022	30,010	32,112	88,692	27,620
Argentina	17,000	244,784	143,991	21,596	327,132	151,478	22,671	337,487	148,863
Republic of Korea	20,109	428,085	212,882	20,918	350,661	167,636	21,775	309,211	142,003
Angola	22,000	182,303	82,865	147,813	982,588	66,475	178,272	1,680,146	94,246
Côte d'Ivoire	23,639	50,824	21,500	24,434	45,580	18,654	28,981	52,232	18,023
Chad	24,000	62,000	25,833	4,982	28,856	57,921	32,635	217,324	66,592
Ethiopia	24,702	220,000	89,062	53,431	450,697	84,351	52,406	1,755,855	335,048
Cameroon	27,054	178,272	65,895	49,012	266,078	54,288	85,953	495,177	57,610
United States of America	33,590	554,335	165,030	39,214	883,100	225,200	59,370	1,450,250	244,273
Democratic People's Republic of Korea	36,500	490,000	134,247	29,000	390,000	134,483	41,148	561,796	136,531
Zambia	37,000	52,000	14,054	60,638	200,450	33,057	27,341	109,336	39,990
Bangladesh	41,278	383,000	92,786	31,697	305,025	96,232	23,014	235,881	102,495
Guinea	42,695	162,760	38,122	25,000	82,194	32,878	55,014	281,700	51,205
Brazil	43,158	472,422	109,463	42,241	477,472	113,035	57,290	805,412	140,585
Japan	44,500	1,008,000	226,517	40,500	1,026,000	253,333	34,300	748,700	218,280
Democratic Republic of the Congo	49,092	246,000	50,110	48,585	243,460	50,110	108,211	542,686	50,151
Kenya	51,320	543,089	105,824	77,821	1,034,204	132,895	65,973	893,656	135,458
Cuba	53,075	261,000	49,176	78,496	437,100	55,684	54,176	555,078	102,458
Mozambique	55,000	400,000	72,727	86,300	900,000	104,287	33,970	451,204	132,824
Haiti	58,000	172,000	29,655	75,000	294,172	39,223	9,333	42,002	45,004
Ghana	65,000	90,000	13,846	72,249	117,879	16,316	73,621	154,260	20,953
Madagascar	92,000	520,000	56,522	127,000	910,857	71,721	132,579	1,113,142	83,961
Papua New Guinea	102,000	480,000	47,059	120,000	595,000	49,583	145,077	743,735	51,265
Burundi	112,000	734,172	65,551	138,000	926,319	67,125	93,578	1,023,458	109,370
Philippines	132,349	557,386	42,115	114,380	560,516	49,005	83,338	525,862	63,100
India	134,400	1,152,400	85,744	124,300	1,119,700	90,080	110,000	1,156,000	105,091
Indonesia	172,243	1,665,547	96,698	183,874	2,057,913	111,920	86,360	1,806,339	209,164
Rwanda	179,941	862,562	47,936	123,386	803,228	65,099	183,889	1,247,584	67,844
Viet Nam	270,200	1,744,600	64,567	146,600	1,211,300	82,626	116,698	1,402,350	120,169
United Republic of Tanzania	282,020	565,220	20,042	651,940	1,417,390	21,741	539,513	3,921,590	72,688
Uganda	539,000	2,354,000	43,673	463,000	1,943,000	41,965	463,568	1,949,476	42,054
**Nigeria**	817,000	2,451,000	30,000	1,100,000	3,300,000	30,000	1,717,659	4,145,488	24,135
**China**	5,945,975	126,143,701	212,150	3,253,056	70,040,593	215,307	2,373,737	51,992,156	219,031

	1999			2009			2019		
Country	A (Ha)	P (Tons)	Yield (Hg/Ha)	A (Ha)	P (Tons)	Yield (Hg/Ha)	A (Ha)	P (Tons)	Yield (Hg/Ha)
**World**	9,743,043	147,148,709	--	7,844,474	96,364,636	--	7,399,599	85,908,695	--
X	95,520	1,442,634	86,334	76,907	944,751	98,456	72,545	842,242	111,289
SD	594,517	12,477,383	64,060	345,461	6,928,029	73,477	294,571	5,161,618	82,978
Min	14	48	9,947	4	12	8,000	2	2	10,000
Max	5,945,975	126,143,701	301,617	3,253,056	70,040,593	400,000	2,373,737	51,992,156	385,997
Country (Max)	China	China	Palestine	China	China	Senegal	China	China	Senegal

Source (FAOSTAT)		<1,000 Ha		10,000 - 99,999 Ha	
www.fao.org/faostat/en/#data/QC		1,000 - 9,999 Ha		100,000 - 999,999 Ha	

	>1,000,000 Ha
--	No available data
