# Supplementary material for: Sweet Potato (Ipomoea batatas L.) Phenotypes: From Agroindustry to Health Effects
Source: Foods. 2022 Apr 6;11(7):1058. doi: 10.3390/foods11071058 (PMC8997864; doi:10.3390/foods11071058)
Supplement: Supplementary file 1 [file foods-11-01058-s001.zip › Table S2.pdf]

Supplementary Material (Table S2) - Nutrient data for raw/proccesed sweet potatoes and other edible roots & tubers

	Potatoes	Cassava	Yams	Orange-fleshed sweet potato (OFSP)							
	Raw	Raw	Raw	Raw	Leaves	Canned	Canned/mashed	Candy	Chips	Boiled	Fries
NDB # / FDC Id.	*11352*	*11134*	*11601*	*11507*	*11505*	*1103254*	*11514*	*1103252*	*1103258*	*1103247*	*1103257*
Water (g)	79.3	59.7	69.6	77.0	86.8	75.7	73.9	57.5	4.5	79.8	44.5
Energy (Kcal)	77	160	118	86	42	91	101	178	529	76	259
Energy (KJ)	322	667	494	359	175	380	422	743	2207	317	1081
Protein (g)	2.1	1.4	1.5	1.6	2.5	1.6	2.0	1.0	**2.9**	1.4	2.1
Fat (g)	0.1	0.3	0.2	0.1	0.5	0.2	0.2	3.3	32.2	0.1	18.1
Carbohydrates (by difference)	17.5	38.1	27.9	20.0	8.8	21.0	23.2	37.5	56.5	17.7	34.1
Sugars (g)	0.8	1.7	0.5	4.2	0.0	5.0	5.5	29.0	8.8	5.7	12.3
Total dietary fiber (TDF, g)	2.1	1.8	4.1	**3.0**	**5.3**	1.8	1.7	1.8	**8.8**	2.5	**5.5**
Ca (mg)	12.0	16.0	17.0	**30.0**	**78.0**	**22.0**	30.0	20.0	**59.0**	27.0	**50.0**
Fe (mg)	0.8	0.3	0.5	0.6	1.0	0.9	1.3	0.5	2.1	0.7	0.8
Mg (mg)	23.0	21.0	21.0	**25.0**	**70.0**	22.0	**24.0**	13.0	**65.0**	18.0	**25.0**
P (mg)	57.0	27.0	55.0	47.0	**81.0**	49.0	52.0	23.0	**144.0**	32.0	56.0
K (mg)	425.0	271.0	816.0	337.0	508.0	311.0	210.0	165.0	**920.0**	229.0	391.0
Na (mg)	6.0	14.0	9.0	55.0	6.0	207.0	75.0	142.0	227.0	181.0	140.0
Zn (mg)	0.30	0.34	0.24	0.30	**0.90**	0.18	0.21	0.15	**0.53**	0.20	0.36
Cu (mg)	0.11	0.10	0.18	0.15		0.14	**0.28**	0.07	**0.41**	0.09	0.17
Se (µg)	0.40	0.70	0.70	0.60	**0.90**	0.70	**0.80**	0.30	**2.10**	0.20	0.40
Ascorbate (C, mg)	19.70	20.60	17.10	2.40	11.00	**26.30**	5.20	9.10	0.00	12.70	6.80
Tiamin (B1, mg)	0.08	0.09	0.11	0.08	**0.16**	0.04	0.03	0.04	0.09	0.06	0.08
Riboflavin (B2, mg)	0.03	0.05	0.03	0.06	**0.35**	0.06	0.09	0.04	**0.16**	0.05	0.09
Niacin (B3, mg)	1.06	0.85	0.55	0.56	**1.13**	0.74	0.96	0.38	**2.10**	0.54	
Piridoxine (B6, mg)	0.30	0.09	0.29	0.21	0.19	0.19	0.24	0.14	**0.53**	0.16	0.17
Folic acid (B9, µg)	15.00	27.00	23.00	11.00	1.00	17.00	11.00	4.00	**37.00**	6.00	**29.00**
Choline (mg)	12.10	23.70	16.50	12.30		12.90		8.00	**36.00**	10.80	**24.90**
A (IU)	2.00	13.0	138.0	**14187.0**	**3778.0**	**7951.7**	**8698.3**	**11221.7**	**23556.7**	**1576.7**	**8300.0**
A (RAE, µg)	0.00	1.0	7.0	**709.0**		**397.0**	**435.0**	**574.0**	**1178.0**	**784.0**	**45.0**
β-carotene (µg)	1.00	8.0	83.0	**8509.0**	**2217.0**	**4771.0**	**5219.0**	**6733.0**	**14134.0**	**9406.0**	**4980.0**
α-carotene (µg)	0.0	0.0	0.0	**7.0**	**42.0**	0.0	0.0	0.0	0.0	0.0	0.0
β-criptoxanthin (µg)	0.0	0.0	0.0	0.0	**58.0**	0.0	0.0	0.0	0.0	0.0	0.0
α-tocopherol (E, mg)	0.01	0.19	0.35	0.26		**1.00**	**1.10**	**1.03**	**9.77**	**0.94**	0.00
Phylloquinone (K, µg)	2.00	1.90	2.30	1.80	**302.20**	0.00	2.40	**4.40**	**24.40**	2.10	**15.70**
Saturated fatty acids (g)	0.025	0.074	0.037	0.018	0.110	0.041	0.040	0.906	2.930	0.030	2.420
16:0 (g)	0.016	0.069	0.034	0.018	0.100	0.040	0.040	0.477	1.390	0.030	1.710
18:0 (g)	0.004	0.005	0.004	0.000	0.000	0.000	0.000	0.200	0.000	0.000	0.000
Monounsaturated fatty acids (g)	0.002	0.075	0.006	0.001	0.020	0.008	0.008	**1.143**	**9.100**	0.000	**7.550**
18:1 (g)	0.001	0.075	0.006	0.001	0.020	0.008	0.008	**1.116**	**8.800**	0.000	**7.380**
Polyunsaturated fatty acids (g)	0.042	0.048	0.076	0.014	**0.228**	0.084	0.080	**0.994**	**12.160**	0.060	**6.680**
18:2 (g)	0.032	0.032	0.064	0.013	**0.192**	**0.071**	**0.070**	**0.882**	**11.020**	0.060	**5.874**
18:3 (g)	0.010	0.017	0.012	0.000	0.000	0.013	0.000	**0.112**	**1.140**	0.000	**0.796**

USDA Nutrient Data Bank: https://fdc.nal.usda.gov/; Storage root or tuber (R/T)
