# Supplementary figures and images for: Sweet Potato (Ipomoea batatas L.) Phenotypes: From Agroindustry to Health Effects
Source: Foods. 2022 Apr 6;11(7):1058. doi: 10.3390/foods11071058 (PMC8997864; doi:10.3390/foods11071058)

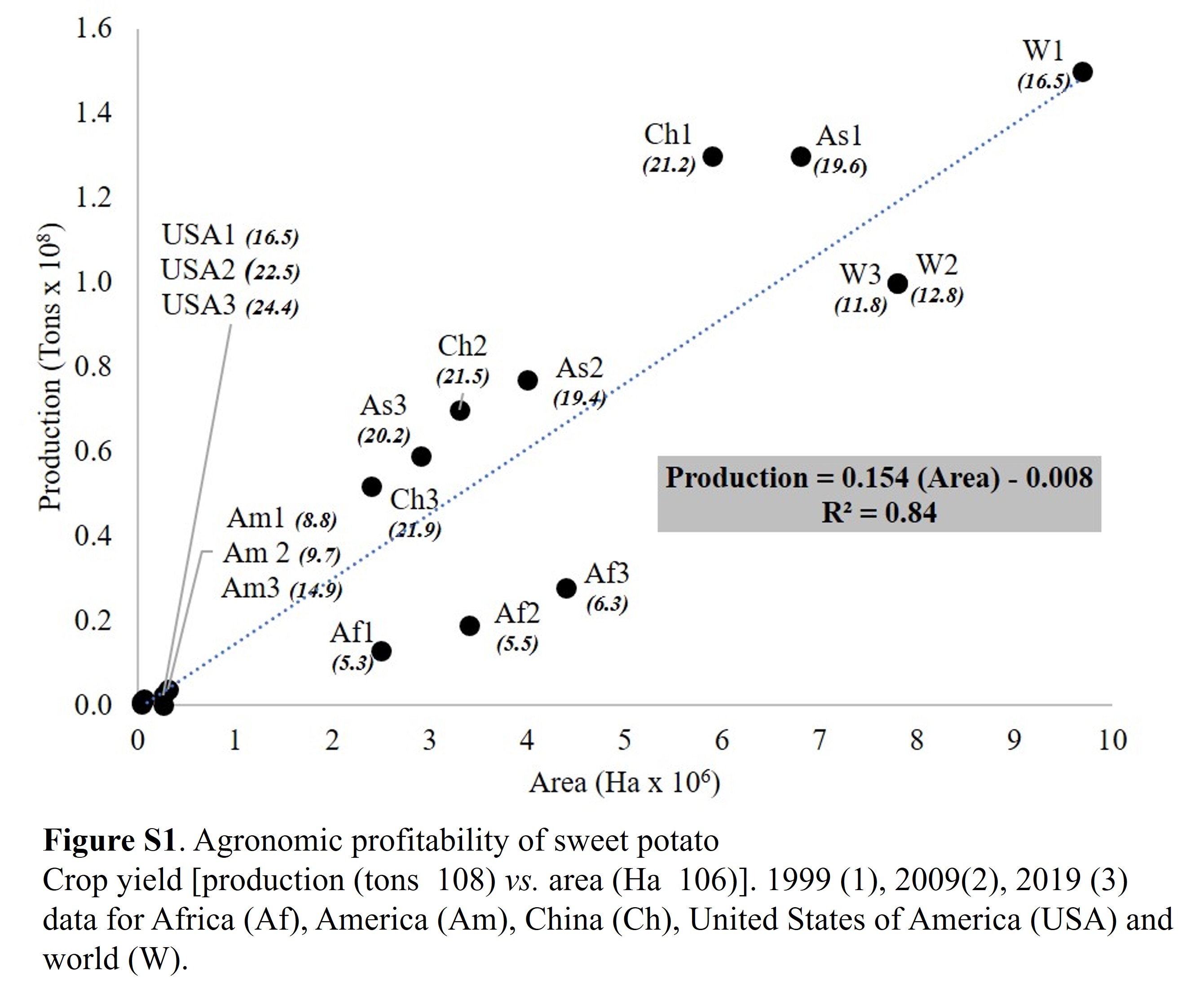

Supplement: Supplementary file 1 [file foods-11-01058-s001.zip › Figure S1.jpg]
